# The Efficacy of Therapies for Phantom Limb Pain in Amputees Assessed via Functional Magnetic Resonance Imaging (fMRI) and Electroencephalography (EEG) Measures of Cortical Reorganization: A Scoping Review

**DOI:** 10.7759/cureus.113810

**Published:** 2026-08-02

**Authors:** Caitilin Montgomery, Kayln Pate, Elena L Richardson, Harvey N Mayrovitz

**Affiliations:** 1 Osteopathic Medicine, Nova Southeastern University Dr. Kiran C. Patel College of Osteopathic Medicine, Fort Lauderdale, USA; 2 Medical Education, Nova Southeastern University Dr. Kiran C. Patel College of Allopathic Medicine, Davie, USA

**Keywords:** amputation, cortical reorganization, electroencephalography, functional magnetic resonance imaging, mirror therapy, neuroplasticity, phantom limb pain, transcranial direct stimulation, transcranial magnetic stimulation

## Abstract

Phantom limb pain (PLP) is a prevalent and debilitating condition affecting most amputees, attributable to complex maladaptive neuroplastic changes of the peripheral and central nervous system components. Cortical reorganization, particularly within the primary motor (M1) and somatosensory (S1) cortices, has been proposed as the central phenomenon underlying PLP. Neuroimaging modalities such as functional magnetic resonance imaging (fMRI) and electroencephalography (EEG) provide objective measures for evaluating these neuroplastic changes and assessing treatment efficacy. This scoping review aims to evaluate nonpharmacologic therapies for PLP and to obtain objective, evidence-based measures of clinical benefit. Three databases (Embase, Ovid MEDLINE, and Web of Science) were searched for peer-reviewed English-language articles published between 2015 and 2025 that included fMRI and EEG measurements to determine the efficacy of treatments in upper- and lower-extremity amputees. Search terms included "amputation," "cortical reorganization," "rehabilitation," "therapy," "treatment," and "phantom limb." A total of 565 articles were initially identified. After removing duplicates, 392 articles were screened, 39 full texts reviewed for eligibility, and seven determined to meet the inclusion criteria. The findings suggest that effective interventions were generally associated with changes in cortical organization of M1 and S1 regions. Mirror therapy (MT) trials showed consistent reductions in PLP, accompanied by normalization of maladaptive sensorimotor activity. Brain stimulation techniques exhibited strong neuromodulatory effects, with significant pain reduction and corresponding changes in cortical excitability and thalamocortical networks. Prosthetic use and augmented reality (AR) therapies demonstrated functional cortical adaptations, though their correlation with pain reduction was less consistent. Overall, many studies demonstrated parallel improvements in pain and cortical reorganization. It is concluded that nonpharmacologic therapies for PLP are frequently associated with measurable changes in cortical organization, particularly within the M1 and S1 cortices, highlighting the role of neuroplasticity in both the pathogenesis and treatment of PLP. However, the relationship between cortical changes and PLP reduction remains inconsistent, suggesting that PLP is a multifactorial, network-level condition involving primary, secondary, and subcortical regions. These findings highlight the potential of fMRI and EEG as objective measures of treatment response and their role as potential biomarkers. This scoping review reinforces the need for further research to better define mechanisms and optimize targeted, multimodal treatment strategies.

## Introduction and background

Post-amputation sensations are a well-documented phenomenon and include phantom limb pain (PLP), residual limb (stump) pain, phantom limb sensations, and telescoping phenomena [1]. Among these, PLP is a common and clinically significant chronic condition, affecting approximately 82% of patients within the first year following amputation [1]. PLP is characterized by painful sensations perceived in the absent limb, often described as burning, stabbing, aching, or electric in nature [2]. In contrast, phantom limb sensations are typically nonpainful and may include feelings of movement, position, or presence of the missing limb, making this distinction important for appropriate diagnosis and management [3]. Most patients who develop PLP report onset within the first month after amputation, with a secondary peak in incidence around one year postoperatively [1,3]. These symptoms can be persistent and debilitating, significantly impacting functional outcomes and overall quality of life.

Numerous mechanisms have been proposed to underlie the pathogenesis of PLP, most of which involve maladaptive changes at the peripheral, spinal, and central levels. Peripheral mechanisms include aberrant nerve regeneration and increased expression of nociceptive fibers, while central mechanisms are largely explained through principles of neuroplasticity, particularly reorganization within the primary somatosensory (S1) and primary motor cortex (M1) [4]. However, the literature remains inconsistent regarding which forms of cortical reorganization serve as the primary neurophysiological basis of PLP. Some studies in upper-extremity amputees demonstrate that greater degrees of sensorimotor cortical reorganization are significantly correlated with increased PLP severity [5], whereas others report no clear relationship between motor cortex reorganization and pain intensity [3]. Some work suggests that these discrepancies may be methodological, as observed associations between M1/S1 reorganization and PLP vary depending on how cortical changes are quantified [6]. Similarly, studies of lower-extremity amputations demonstrate M1/S1 reorganization, but with weaker or non-significant correlations with PLP intensity [7].

Due to the overall low evidence of most trials, the best first-line therapy for PLP is controversial. Many current therapeutic approaches aim to modulate cortical reorganization. Sensory-motor integration therapies, including mirror therapy (MT) and virtual/augmented reality (AR), aim to restore proper connection between motor intention and sensory feedback, resulting in a normalization of cortical representations [8]. Neuromodulation techniques, such as transcranial direct current stimulation (tDCS), deep brain stimulation, and transcranial magnetic stimulation (TMS), directly impact cortical excitability [9]. While these two main categories of therapeutic interventions differ in mechanisms and neurophysiologic outcomes, most are hypothesized to exert therapeutic effects by altering maladaptive neuroplasticity.

Functional magnetic resonance imaging (fMRI) and electroencephalography (EEG) provide real-time observation of cortical changes seen in PLP. fMRI detects dynamic patterns of brain activity by measuring blood-oxygen-level-dependent (BOLD) signals, which reflect the magnetic properties of oxygenated and deoxygenated hemoglobin [10]. The BOLD signal allows fMRI to map cortical activation patterns and functional connectivity. In contrast, EEG provides high temporal resolution and insight into cortical excitability and neural network activity [2]. Both imaging modalities offer potential objective biomarkers for therapeutic response that may supplement traditional subjective pain assessments.

The rise of fMRI and EEG as tools for evaluating cortical reorganization in PLP warrants a structured mapping of the existing evidence. This scoping review aims to characterize the range of therapeutic interventions assessed using fMRI and/or EEG, summarize how cortical reorganization is objectively measured across studies, and identify gaps in the current literature. Therefore, this scoping review aims to synthesize the available fMRI and EEG evidence to examine the proposed relationship between cortical reorganization and PLP and how therapeutic interventions influence these neuroplastic changes and associated clinical outcomes.

## Review

Study design and protocol

This scoping review was conducted using the Joanna Briggs Institute (JBI) methodology for scoping reviews and adheres to the Preferred Reporting Items for Systematic reviews and Meta-Analyses extension for Scoping Reviews (PRISMA-ScR) guidelines [11,12]. No review protocol was registered prior to conducting this study.

Search Strategy

A base search strategy was developed through analysis of key terms, and searches were conducted in Embase, Ovid MEDLINE, and Web of Science. Data collection took place in October 2025, and the base search was utilized for each database. The search terms "amputation," "cortical reorganization," "rehabilitation," "therapy," "treatment," and "phantom limb" were entered into the controlled descriptors for each database. The Boolean operator AND was used for simultaneous occurrences and OR for their synonyms. The full search strategy can be found in Table 1.

**Table 1:** Database search results exp: exploded subject heading search; fMRI: functional magnetic resonance imaging; mp: multipurpose search field; MRI: magnetic resonance imaging; TMS: transcranial magnetic stimulation Search strategies were adapted to the indexing structure and syntax requirements of each database. Boolean operators (AND, OR) were used to combine concepts. “exp” indicates inclusion of all narrower indexed subject headings beneath a broader term. “mp” indicates a multi-purpose search across titles, abstracts, subject headings, keywords, and supplementary concept fields. Search results reflect the number of articles retrieved during database querying, prior to duplicate removal and screening

Database	Search number	Search terms	# of articles
Web of Science	1	phantom pain (All Fields) or phantom limb (All Fields) or phantom limb pain (All Fields) or stump pain (All Fields) or residual limb pain (All Fields)	6,988
	2	functional magnetic resonance (All Fields) or functional magnetic resonance imaging (All Fields) or fMRI (All Fields) or functional MRI (All Fields)	190,224
	3	cortical reorganization (All Fields) or neuroplasticity (All Fields) or cortical plasticity (All Fields) or cortical reorganisation (All Fields) or deafferentation (All Fields)	47,993
	4	therapy (All Fields) or rehabilitation (All Fields) or noninvasive brain stimulation (All Fields) or transcranial magnetic stimulation (All Fields) or TMS (All Fields) or repetitive transcranial magnetic stimulation (All Fields) or repetitive TMS (All Fields) or transcutaneous electrical	10,232,001
	5	ALL=(amputation) OR ALL=(limb loss) OR ALL=(stump) OR ALL=(amputee)	95,299
	6	2 OR 3	233,554
		1 AND 4 AND 5 AND 6	373
Embase	1	'amputation'/exp OR ‘leg amputation’ OR 'lower limb loss' OR 'leg stump' OR 'above knee amputation' OR 'below knee amputation' OR 'knee amputation' OR 'foot amputation' OR 'ankle amputation' OR 'partial foot amputation' OR 'toe amputation' OR 'leg amputee' OR 'transtibial amputee' OR 'transfemoral amputee' OR 'forefoot amputation' OR 'hindfoot amputation' OR ‘arm stump’ OR ‘leg stump’ OR ‘experimental amputation’ OR ‘facial amputation’ OR ‘genital amputation’ OR ‘mastectomy’ OR ‘orthopedic amputation’ OR ‘reamputation’ OR ‘traumatic amputation’ OR ‘limb amputation’ OR ‘arm amputation’ OR ‘above elbow amputation’ OR ‘below elbow amputation’ OR ‘elbow amputation’ OR ‘amputation’ OR ‘forelimb amputation’	207,495
	2	'phantom pain'/exp OR 'phantom limb' OR 'stump pain' OR 'residual limb pain'	4,864
	3	'functional magnetic resonance imaging'/exp OR 'cortical reorganization'/exp OR 'cortical reorganization' OR 'nerve cell plasticity' OR 'deafferentation'	225,707
	4	'rehabilitation'/exp OR 'mirror therapy' OR 'transcranial magnetic stimulation' OR 'repetitive transcranial magnetic stimulation' OR 'nerve stimulation' OR 'transcutaneous electrical nerve stimulation' OR 'analgesia' OR 'virtual reality' OR 'graded motor imagery' OR 'targeted muscle reinnervation' OR 'transcranial electrical stimulation'	1,081,365
	5	1+2+3+4	152
MEDLINE	1	exp Amputation/	26,313
	2	("amputation*" or "disarticulation*" or "hemipelvectomy*").mp. [mp=title, book title, abstract, original title, name of substance word, subject heading word, floating sub-heading word, keyword heading word, organism supplementary concept word, protocol supplementary concept word, rare disease supplementary concept word, unique identifier, synonyms, population supplementary concept word, anatomy supplementary concept word]	65,780
	3	(Phantom Limb* or neuralgia* or phantom limb syndrome*).mp. [mp=title, book title, abstract, original title, name of substance word, subject heading word, floating sub-heading word, keyword heading word, organism supplementary concept word, protocol supplementary concept word, rare disease supplementary concept word, unique identifier, synonyms, population supplementary concept word, anatomy supplementary concept word]	39,440
	4	exp Magnetic Resonance Imaging/	583,759
	5	("magnetic resonance imaging*" or "functional magnetic resonance imaging*").mp. [mp=title, book title, abstract, original title, name of substance word, subject heading word, floating sub-heading word, keyword heading word, organism supplementary concept word, protocol supplementary concept word, rare disease supplementary concept word, unique identifier, synonyms, population supplementary concept word, anatomy supplementary concept word]	695,500
	6	Exp Rehabilitation/	385,817
	7	("Mirror movement therapy*" or "rehabilitation*" or "magnetic field therapy*" or "electric stimulation therapy*" or "treatment*" or "therapy*").mp. [mp=title, book title, abstract, original title, name of substance word, subject heading word, floating sub-heading word, keyword heading word, organism supplementary concept word, protocol supplementary concept word, rare disease supplementary concept word, unique identifier, synonyms, population supplementary concept word, anatomy supplementary concept word]	10,507,254
	8	(1 OR 2) AND 3 AND (4 OR 5) AND (6 OR 7)	40

Inclusion Criteria

To be included in the scoping review, articles needed to be peer-reviewed, written in English, and published between 2015 and 2025, involving patients of any age who experienced PLP following amputation of their respective anatomical areas and had completed any type of post-amputation rehabilitation. In addition, cortical reorganization following therapy had to be evaluated using fMRI, EEG, or other neurophysiologic measures of cortical activity.

Exclusion Criteria

Excluded articles were scoping reviews, systematic reviews, case reports, articles based solely on animal research, and conference reports with only abstracts.

Selection Strategy

**Figure 1:**
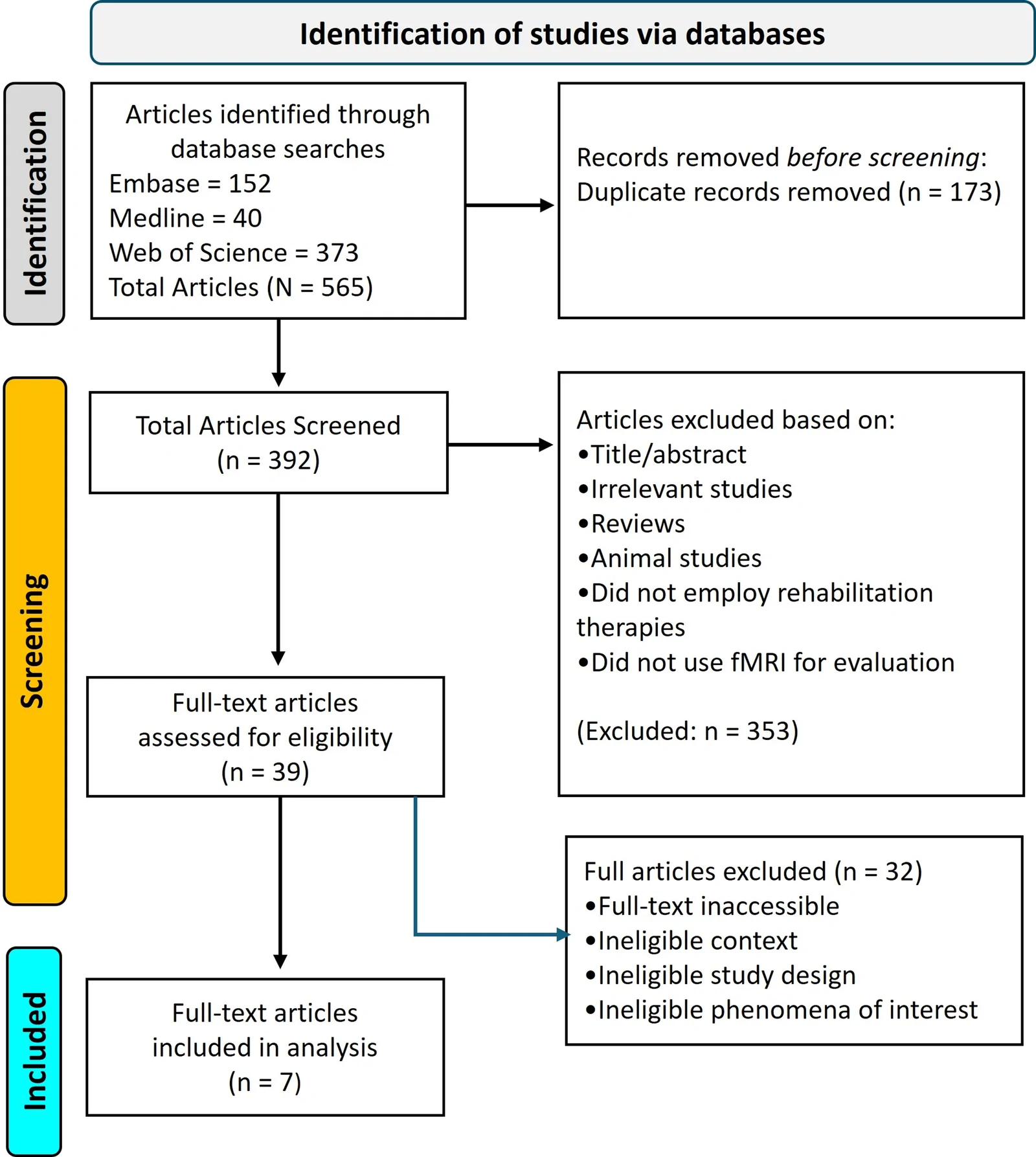
PRISMA flowchart detailing the search and selection process PRISMA: Preferred Reporting Items for Systematic Reviews and Meta-analyses

The articles were screened using Rayyan (Rayyan Systems Inc.; Cambridge, MA, US). Following screening, Google Sheets was used for data extraction, and Google Docs for documenting additional notes and information regarding sources for this review. The search identified 565 citations, of which 173 duplicates were removed using Rayyan, yielding 392 papers for screening. Titles and abstracts were independently screened by three reviewers. Based on the inclusion criteria, 39 articles were deemed relevant for further consideration. A second screening of the full-text articles was then performed by all reviewers, during which each article was evaluated against the inclusion and exclusion criteria. Each reviewer voted "yes" or "no," and articles without unanimous agreement were included or excluded based on majority vote. A total of seven studies met the inclusion criteria and were included in the final review. This process is summarized in the PRISMA diagram (Figure 1).

Results

Seven studies were included in this review that discussed therapeutic interventions targeting PLP and cortical reorganization. Across all seven studies, the effectiveness of treatment was evaluated by comparing the degree of neural activity and cortical reorganization via neuroimaging before and after therapeutic intervention. Five therapies were evaluated: MT, prosthetic use, brain stimulation techniques, augmented reality (AR)-based rehabilitation, and tDCS. Overall, review of these seven articles suggests that successful therapeutic interventions were associated with measurable changes in cortical organization, particularly within M1 and S1 cortices. While M1 and S1 are the primary anatomical areas, other cortical and subcortical areas are also involved in post-amputation pain processing. The included studies were categorized by study aim, sample size, design, main findings, and conclusions.

Mirror Therapy (MT)

MT, one of the most frequently studied therapies for PLP, showed consistent effects on cortical reorganization. Chan et al. report a significant reduction in PLP from Session 1 to Session 3, with a reduction in visual analog scale (VAS) scores [5,13]. With respect to imaging, subjects showed increased visual responsiveness within the S1, especially to foot images, following amputation of a lower limb. This suggests that following amputation, input-deprived cortical areas are more responsive to visual input. Following MT, this enhanced visual responsiveness decreased, suggesting normalization of sensorimotor cortical processing and reorganization. Reduced visual responsiveness correlated with lower VAS scores. These findings support the hypothesis that MT promotes nonmaladaptive reorganization of sensorimotor components underlying PLP. Most importantly, greater baseline responsiveness correlated with increased reduction of PLP, which could point to a potential biomarker of MT pain reduction.

Prosthetic Use

Prosthetic use was evaluated for cortical reorganization associated with the use of a hand prosthesis in upper-limb amputees using fMRI and EEG data [7]. Imaging taken during prosthesis use showed activation in areas of the primary sensorimotor cortex, including regions representing the missing limb, but reduced cerebellar recruitment during phantom hand movement after training. This is consistent with more efficient motor processing and motor learning. Secondly, TMS-EEG measured cortical asymmetry at baseline, characterized by increased alpha-wave frequency and decreased delta-wave frequency in the hemisphere contralateral to amputation following training. The difference in frequency between the two hemispheres was reported to be trending toward significance. Alpha-wave frequencies represent top-down cortical processing, while delta-wave frequencies represent diseased neural tissue and maladaptive plastic changes [7]. Importantly, structural imaging did not demonstrate significant changes, suggesting that prosthesis-induced plasticity is primarily functional rather than anatomical. This supports the previous finding that a decrease in maladaptive changes is represented by a decrease in cerebellar recruitment following prosthetic use. Additionally, Granata et al. [10] discussed a limitation of the study worth noting: cortical activity related to sensory feedback provided by the device.

A related study was performed on cortical plasticity after hand prosthesis use but investigated the reorganization of the sensorimotor cortex corresponding to the amputated limb [10]. The question was whether this area was fully reorganized by neighboring body area representation. Following training, there was a significant increase in motor map area and motor evoked potential (MEP) amplitudes in the affected hemisphere, indicating partial restoration of interhemispheric symmetry. The results of this study were inconclusive regarding complete “cortical invasion.” Changes in motor cortical activation patterns were observed following prosthesis use, including changes in activation, activation magnitude, activation area, laterality differences, and sensorimotor network connectivity compared to baseline. Two participants had PLP reductions, with VAS decreases of 34-39%. However, no direct correlation between motor cortical changes and PLP improvement was observed, suggesting that PLP reduction and motor reorganization may represent separate processes and that PLP is likely mediated by more complex, multilevel mechanisms beyond the M1 [10].

Thalamic Deep Brain Stimulation

Abreu et al. [1] evaluated the long-term efficacy of thalamic deep brain stimulation (DBS) for posttraumatic neuropathic limb pain. Clinical outcomes were assessed using the VAS, Brief Pain Inventory (BPI), University of Washington Neuropathic Pain Scale (UWNPS), and the 36-Item Short Form Health Survey (SF-36) [13,14-17]. Across the study population, DBS resulted in a statistically significant overall VAS improvement of 76.4%. Among patients with post-amputation pain, the median VAS improvement at five years was 90%, compared with 75% among patients with brachial plexus pain. Pain relief was maintained throughout long-term follow-up, with no significant difference between the three- and five-year assessments. MRI analyses demonstrated changes in thalamocortical circuitry involved in sensory processing and pain perception. Within the DBS cohort, patients with greater overlap of stimulated tissue and the ventral posterolateral (VPL) nucleus achieved greater pain reduction (67% VAS improvement) than those with minimal VPL recruitment (50.6% VAS improvement) [1]. These findings suggest that targeting the VPL nucleus may contribute to improved modulation of central pain pathways.

Augmented Reality (AR)

Thøgersen et al. [18] evaluated individualized AR therapy. In this study, AR was used therapeutically to create the illusion of the missing limb for the subject. After two weeks of AR therapy, acquired fMRI data during the lip-pursing task showed a significant decrease in activity of the S1, representative of and contralateral to the amputation site. Activation of Brodmann area 3b was observed both before and after treatment, but activation in this region was reduced following the intervention. The location of the cortical representation of the lip in S1 shifted an average of 4.62 mm toward the hemisphere associated with the intact limb, approaching statistical significance. The retraction of lip-representative areas back to pre-amputation organization was observed concurrently with reductions in PLP. The nature of sensation post-AR therapy was also discussed. The study also evaluated subjective experiences associated with the virtual limb, including telescoping (the perception that the phantom limb has shortened or retracted toward the residual limb), embodiment (the extent to which the virtual limb is experienced as part of one's own body), ownership (the feeling that the virtual limb belongs to the individual), location (the perceived spatial position of the phantom or virtual limb), and agency (the sense of voluntary control over the virtual limb). Pain outcomes were assessed using the Numeric Rating Scale (NRS) [19] and the McGill Pain Questionnaire (MPQ) [20]. Although this study revealed no significant changes in these sensations post-AR therapy, there were visible trends in agency and embodiment throughout the course of the study.

Transcranial Direct Current Stimulation (tDCS)

Gunduz et al. [21] evaluated tDCS alongside MT. In this study, tDCS was administered for two weeks within a four-week MT intervention period for select lower-limb amputee subjects. Results showed overall improvement in PLP, with a mean VAS reduction of 2.64 points, and active tDCS produced a statistically significant reduction in PLP compared with sham tDCS. PLP reduction following active tDCS was associated with increased intracortical inhibition (coefficient = 0.96, P = 0.02) in the affected hemisphere, with SICI (short-interval intracortical inhibition) changes specifically correlating with pain reduction. Increased intracortical facilitation (ICF) was also measured in correlation with direct tDCS, the level of ICF being measurable to its representative analgesic value. Gunduz et al. [21] also reported a posterolateral shift in the cortical map center of gravity in the active tDCS group. Hierarchical regression models showed no statistically significant interaction between tDCS and MT, indicating that the effects of each intervention were independent. MT did not significantly reduce PLP and did not induce changes in cortical excitability or cortical mapping. Additionally, pain reduction was not mediated by changes in depression, anxiety, or other secondary outcomes, suggesting a direct neuromodulatory effect of tDCS on motor cortex pain pathways.

In a study discussing tDCS on selected upper-limb amputees, Kikkert et al. [22] showed that tDCS therapy yielded significant reductions in PLP with effects lasting up to one week following a single administration. This study further discussed that PLP improvement was associated with decreased phantom hand activity in the S1/M1 missing hand cortex, with preceding activation in the insula and secondary somatosensory cortex (S2) during stimulation. This suggests a collaborative mechanism of pain modulation, including the M1, secondary, and subcortical areas.

Summary

Seven studies were included in this review that discussed therapeutic interventions targeting PLP and cortical reorganization. Across all seven studies, the effectiveness of treatment was evaluated by comparing the degree of neural activity and cortical reorganization via neuroimaging before and after therapeutic intervention. Five therapies were evaluated: MT, prosthetic use, brain stimulation techniques, AR-based rehabilitation, and tDCS. Overall, review of these seven articles suggests that successful therapeutic interventions were associated with measurable changes in cortical organization, particularly within M1 and primary S1 cortices. While M1 and S1 are the primary anatomical areas, other cortical and subcortical areas are also involved in post-amputation pain processing. The included studies were categorized by study aim, sample size, design, main findings, and conclusions (Table 2).

**Table 2:** Data extraction of studies examining cortical reorganization and therapeutic interventions in PLP AR: augmented reality; BPI: Brief Pain Inventory; DBS: deep brain stimulation; eVAT: electric volume of activated tissue; EMG: electromyography; fMRI: functional magnetic resonance imaging; ICF: intracortical facilitation; M1: primary motor cortex; McGill: McGill Pain Questionnaire; MEP: motor-evoked potential; MT: mirror therapy; NPSI: Neuropathic Pain Symptom Inventory; NRS: Numeric Rating Scale; NS: not statistically significant; PLP: phantom limb pain; S1: primary somatosensory cortex; S2: secondary somatosensory cortex; SF-36: 36-Item Short Form Health Survey; SICI: short-interval intracortical inhibition; tDCS: transcranial direct current stimulation; TMS: transcranial magnetic stimulation; UWNPS: University of Washington Neuropathic Pain Scale; VAS: visual analog scale; VPL: ventral posterolateral nucleus of the thalamus; VR: virtual reality; VTA: volume of tissue activated; EEG: electroencephalography Percent reductions indicate improvement in pain severity unless otherwise specified. Cortical reorganization findings were assessed using neuroimaging and/or neurophysiologic techniques including fMRI, EEG, and TMS motor mapping. NS indicates findings that did not reach statistical significance

Authors	Aims	Study population and sample size	Methodology	Key findings	Limitations	Therapy
Abreu et al., 2022 [1]	To evaluate long-term (5-year) efficacy of thalamic DBS for neuropathic limb pain and correlate outcomes with neuroimaging (VTA/eVAT)	16 enrolled; 15 implanted; 14 with 5-year follow-up. Includes 6 phantom limb pain and 10 brachial plexus injury patients	Prospective, open-label case series. VPL thalamic DBS with clinical outcomes (VAS, UWNPS, BPI, SF-36) and imaging-based VTA/eVAT analysis	Significant long-term pain reduction at 5 years (VAS -76.4%, p = 0.0001). BPI improved 65.1% (p = 0.0505); UWNPS 35.2% (NS); SF-36 minimal change (5%, NS). Greater improvement in PLP (-90%) vs brachial plexus injury (-75%). Effects were sustained from years 3–5. Better outcomes were associated with greater VPL engagement, though meaningful relief occurred without direct overlap	Small sample, no control group, incomplete imaging data	tDBS
Chan et al., 2019 [5]	To investigate neural correlates of mirror therapy in amputees with phantom limb pain (PLP), focusing on whether visual responsiveness in the sensorimotor cortex changes over the course of treatment and relates to pain reduction	9 unilateral lower-limb amputees with ongoing PLP and 9 matched controls without amputation	Prospective longitudinal study using fMRI to evaluate cortical changes before, during, and after a 4-week mirror therapy intervention in unilateral lower-limb amputees with phantom limb pain. Pain was assessed using the VAS	Mirror therapy reduced PLP by 46% (VAS, Session 1 to 3). At baseline, amputees showed increased visual responses to limb images in the sensorimotor cortex, especially for feet; this was not seen in controls. After 4 weeks, visual responsiveness decreased in sensorimotor, extrastriate, and parietal regions (not SII), paralleling pain reduction. Higher baseline visual responsiveness predicted greater PLP improvement	Small sample size; lower-limb amputees only; no amputee comparison group receiving alternative treatment; variability in amputation characteristics and medication use; limited power for subgroup analyses; findings are preliminary and need replication	Mirror therapy
Granata et al., 2020 [7]	To assess whether prolonged use of a bidirectional sensorized hand prosthesis induces functional changes in sensorimotor brain networks (at rest and during tasks) in upper-limb amputees, and to inform development of fully integrated prosthetic limbs	5 consecutive left trans-radial amputees with heterogeneous clinical characteristics (time since amputation, prosthesis use, baseline PLP variability); 4 reported PLP at baseline, and 1 was pain-free	Longitudinal interventional study in 3 trans-radial amputees over 6 months of training with a bidirectional sensory-feedback prosthesis. Outcomes included TMS motor mapping and PLP measures (VAS, NPSI), with pre-post comparisons of motor cortex reorganization and pain	Long-term use of a sensorized prosthesis led to functional (not structural) brain changes. EEG showed increased alpha and decreased delta power. Resting-state fMRI showed improved sensorimotor connectivity, while the default mode network was unchanged. Task fMRI showed increased contralateral M1 activation and reduced cerebellar recruitment, suggesting more efficient motor processing. TMS-EEG asymmetry persisted and was not fully normalized	Very small, heterogeneous sample (amputation history, PLP severity, prosthesis type, training duration). Protocols were not uniform and not all participants completed all measures. No control group, limiting generalizability. Findings are preliminary but suggest possible functional brain adaptation with sensorized prosthesis use	Prosthesis
Granata et al., 2020 [10]	To evaluate motor cortex plasticity following training with a bidirectional hand prosthesis and assess its relationship with changes in phantom limb pain (PLP)	3 adult transradial upper limb amputees (2 with PLP, 1 without)	This longitudinal interventional study evaluated 3 transradial amputees over a 6-month training period using a bidirectional hand prosthesis with sensory feedback. Cortical reorganization was assessed using transcranial magnetic stimulation (TMS) motor mapping, while phantom limb pain (PLP) was measured using the Visual Analogue Scale (VAS) and Neuropathic Pain Symptom Inventory (NPSI). Pre- and post-intervention comparisons were conducted to assess changes in motor cortex representation and pain outcomes	At baseline, participants demonstrated reduced motor cortex representation of stump muscles compared to the intact limb. Following training, there was a significant increase in motor map area and motor evoked potential (MEP) amplitudes in the affected hemisphere, indicating partial restoration of interhemispheric symmetry. Two participants with baseline PLP experienced significant pain reductions (VAS ↓ 34-39%, NPSI ↓ 32-55%). However, no direct correlation was found between motor cortical changes and PLP improvement, suggesting that PLP reduction may occur independently of motor cortex reorganization	The study is limited by a very small sample size (n = 3), lack of a control group, and participant heterogeneity in terms of pain status, duration of amputation, and medication use. Additionally, the small sample size limits the ability to establish correlations between cortical changes and PLP, and no functional behavioral outcomes were assessed	Prosthesis
Thøgersen et al., 2020 [18]	To determine whether a 2-week individualized augmented reality (AR) intervention could reduce phantom limb pain (PLP) in amputees with telescoped phantoms, and whether pain improvement would be accompanied by changes in cortical reorganization measured with fMRI. The study also aimed to assess changes in telescoping, embodiment, ownership, location, and agency over the course of treatment	Adults with unilateral upper-limb amputation and chronic PLP. (>2 years, biweekly pain, >20% telescoping). 8 participants enrolled; 7 completed. 6 included in main fMRI, and 5 in peak-shift analyses due to imaging quality	Prospective pre-post pilot (4 weeks): week 1 baseline, weeks 2-3 AR intervention, week 4 assessment, with 1-month follow-up. Participants completed 8 supervised home-based AR sessions (45 min each) using a custom EMG-controlled virtual phantom hand matched to the perceived limb. Tasks included pick-and-place, imitation, and sorting. Outcomes: daily NRS pain diary, McGill pre/post sessions, multidimensional pain inventory, telescoping, embodiment/agency, and pre/post fMRI (lip-pursing task to assess S1 reorganization)	Pain decreased over time (-32% overall; -41% in responders, NRS). McGill scores fell 52% from early to late sessions (p = .032), suggesting cumulative effects. fMRI showed reduced contralateral S1 activity and a ~4.6 mm shift of lip representation toward normal; both correlated with lower PLP. Telescoping improved and tracked with PLP. Embodiment/agency did not change, but higher embodiment was associated with less pain	Small sample (n = 7); no control group; incomplete fMRI data; short follow-up; one participant used VR instead of AR; limited generalizability (upper-limb amputees only)	AR
Gunduz et al., 2021 [21]	To assess the effects of transcranial direct current stimulation (tDCS) and mirror therapy (MT), alone and combined, on phantom limb pain (PLP) in traumatic lower-limb amputees, and to determine whether treatment effects were associated with motor cortex plasticity changes measured by TMS	112 traumatic lower-limb amputees with chronic PLP were randomized into four intervention arms combining active/sham tDCS and active/covered MT (n = 27-29 per group)	Large randomized, blinded, sham-controlled, 2 × 2 factorial trial across 2 sites. MT or covered MT was given for 4 weeks (20 sessions) and active or sham tDCS over contralateral M1 for 2 weeks (10 sessions). Primary outcome: PLP on VAS at 4 weeks. Motor cortex plasticity was assessed with TMS, including cortical excitability and motor mapping	Active tDCS significantly reduced PLP compared to sham (VAS ↓ 2.64 points overall; β = −0.99, P = .04), with no interaction with MT, indicating independent effects. MT did not significantly reduce PLP. tDCS increased intracortical inhibition (SICI) (p = .02) and facilitation (ICF) (p = .03) and produced a posterolateral cortical map shift. PLP reduction correlated with SICI changes, suggesting a motor cortex–mediated mechanism. No effects were seen on mood, and MT produced no significant cortical changes	Only traumatic lower-limb amputees were included, limiting generalizability to upper-limb, congenital, oncologic, or nontraumatic amputees. MT could not be fully double-blinded because of its behavioral nature. TMS was performed over the hand area rather than the leg area, which may limit spatial specificity for lower-limb amputation. Follow-up was relatively short compared with long-term neuromodulation studies. Phenotyping/predictor analyses were exploratory	tDCS
Kikkert et al., 2019 [22]	To investigate neural mechanisms of phantom limb pain (PLP) and how task-concurrent noninvasive brain stimulation (tDCS) produces pain relief	17 upper-limb amputees with PLP (15 completed study) and 15 controls	Within-subject, double-blind, sham-controlled study. Anodal tDCS over S1/M1 during phantom hand movement, with fMRI during and after stimulation and longitudinal pain tracking (up to 1 week)	Single-session tDCS reduced PLP by ~30-50% vs sham, with effects lasting up to 1 week. Pain relief was linked to decreased S1/M1 activity; higher baseline S1/M1 activity was associated with worse PLP. Greater insula and S2 activation during stimulation predicted both S1/M1 downregulation and pain relief. Amputees also showed increased baseline S1/M1–insula connectivity, supporting a network-level mechanism	Small sample; heterogeneity in amputation characteristics; short-term intervention (single session); limited generalizability; mechanistic findings are correlational; some participants excluded/incomplete data	tDCS

Discussion

This review investigates therapeutic interventions for PLP and their consistent association with anatomical and functional changes in cortical reorganization, mainly in the M1 and S1. In addition to S1/M1, a few studies alluded to secondary subcortical involvement in PLP neurophysiology. Although this review includes a limited number of studies with heterogeneous characteristics, consistent patterns provide insight into the neurophysiological mechanisms of PLP, both what has been recorded and what warrants further inquiry. Across the therapies discussed, reductions in pain are generally associated with normalization of maladaptive cortical reorganization, supporting the consensus that therapies targeting alterations in sensorimotor stimuli contribute significantly to reduction in PLP [5,7,10,21-22].

Studies using MT provide the most direct evidence supporting the relationship between PLP and primary cortical reorganization. In part, this is based on the fact that after amputation, input-deprived regions of S1 and M1 show increased responsiveness to visual stimuli. Subjects with greater baseline responsiveness in these regions showed larger reductions in PLP by the end of the MT trial, with concurrent decreases in visual responsiveness within S1 [5]. This suggests that MT plays a role in normalizing maladaptive cortical plasticity and that the degree of baseline activity may serve as a future biomarker indicating individual success and pain reduction following MT rehabilitation [5]. However, not all studies reported consistent findings; for example, Gunduz et al. [21] found that MT alone did not significantly reduce PLP or alter cortical excitability, which may reflect differences in amputation level (upper- versus lower-limb), treatment dose, study design, blinding, or the use of sham-controlled interventions. AR studies also demonstrated reductions in phantom pain that corresponded with decreased activity in S1, specifically within Brodmann area 3b. These findings are consistent with the concept that a reduction in maladaptive neuroplasticity in primary cortical areas coincides with a reduction in PLP [5,18]. The combined evidence is consistent with the hypothesis that the neurophysiological explanation of PLP includes increased reorganization in S1/M1 following amputation.

Studies using neuromodulation approaches to PLP therapy, such as tDCS, have examined the relationship between intracortical inhibition and intracortical facilitation [21]. Intracortical facilitation, as assessed by glutamatergic activity in the motor cortex, increased with stimulation, and the analgesic effect was directly proportional to the amount of increase [21]. Contrastingly, intracortical inhibition, reflecting enhanced gamma-aminobutyric acid (GABA)-ergic activity, increased during stimulation and was associated with pain reduction. This observed interrelationship between excitatory and inhibitory signaling may point to dysregulated cortical and subcortical excitability as a contributing factor in PLP pathophysiology.

Brain stimulation studies demonstrate that modulation of thalamocortical networks, particularly involving the VPL nucleus of the thalamus, is associated with significant improvements in PLP. Greater overlap of stimulated regions within this area was directly correlated with increased pain reduction [1]. This is consistent with the idea that central sensory processing in the thalamus may modulate pain perception, a concept relevant to PLP therapeutic interventions and relief [1,22]. Additionally, the insula, cerebellum, and secondary cortical areas are found to contribute to pain neuromodulation during PLP therapeutic interventions [22]. Together, these findings suggest that PLP neurobiological mechanisms and their respective therapeutic targets are not limited to primary cortical areas and may point to a more complex, distributed network involving interactions among primary, secondary, and subcortical regions [1,22].

Several gaps in the current literature with potentially important implications for the future of PLP therapy have been identified. As previously discussed, input-deprived cortical regions that demonstrate increased responsiveness following amputation may serve as potential biomarkers for predicting response to MT [5]. However, the proposed network-based model reinforces that effective treatment of PLP may require targeting multiple levels of neural processing, rather than focusing on primary cortical reorganization alone [1,21,22]. To further support this approach, additional research is needed to better understand the effects of subcortical neural changes on primary cortical reorganization, not only regarding underlying neurophysiology but also how these mechanisms can be targeted to reduce pain in this patient population.

Limitations

This scoping review has several limitations that should be considered when interpreting its findings. First, and most noticeably, the number of papers included in this review is relatively low. This may reflect the exclusion of non-English literature, the limitation to publications within the past 10 years, the scoping nature of the review (which prioritizes mapping over quantitative synthesis), and potential gaps in the search strategy. This limitation is further mirrored within the included studies themselves, many of which are characterized by small sample sizes and limited subject populations. Secondly, the heterogeneity across studies (including variation in study design, patient populations, interventions, imaging modalities, and outcome measures) makes direct comparison difficult and limits the ability to properly synthesize data. As a result, the findings of this review are largely descriptive rather than quantitative. Additionally, prior literature on PLP has often relied on more subjective measures, such as correlations with visual analog scale scores, without consistently accounting for underlying structural or cortical changes. This variability further complicates efforts to draw definitive conclusions regarding mechanisms and treatment efficacy [2,9].

## Conclusions

PLP is proposed to be a complex, network-level disorder requiring a multimodal therapeutic approach targeting primary, secondary, and subcortical regions. MT and AR are effective in improving pain at the level of the primary cortex. Neuromodulation approaches based on tDCS are also effective at alleviating pain by addressing dysregulation of neuronal excitation and inhibition at both primary and secondary cortical levels. Brain stimulation that affects subcortical pathway activity correlates with pain reduction. While targeting reorganization in primary cortical areas has consistently been shown to correlate with reductions in PLP, further research is needed to better understand subcortical remapping as a potential mechanism and therapeutic target.
